# Inulin Protects Caco‐2 Cells Against Lipopolysaccharide‐Induced Epithelial Barrier Dysfunction

**DOI:** 10.1002/fsn3.70046

**Published:** 2025-03-24

**Authors:** A. Georgetta Skinner, Abdul Malik, M. Rizwan Siddiqui, Vineet Singh, Suhail Akhtar

**Affiliations:** ^1^ Department of Biochemistry, Kirksville College of Osteopathic Medicine A.T. Still University Kirksville Missouri USA; ^2^ Department of Pharmaceutics, College of Pharmacy King Saud University Riyadh Saudi Arabia; ^3^ Department of Biological Sciences Murray State University Murray Kentucky USA; ^4^ Thomas F. Frist, JR. College of Medicine Belmont University Nashville Tennessee USA

**Keywords:** Caco‐2 cells, inflammation, inulin, LPS, tight junction proteins

## Abstract

Lipopolysaccharide (LPS), a component of the outer membrane of Gram‐negative bacteria, triggers inflammatory responses in intestinal epithelial cells. This activation leads to the production of pro‐inflammatory cytokines which disrupt cellular homeostasis. LPS also impairs the integrity of the intestinal epithelial barrier by downregulating tight junction proteins, resulting in increased intestinal permeability. This compromised barrier function can allow further translocation of luminal antigens, perpetuating inflammation and contributing to gut‐related disorders such as inflammatory bowel disease (IBD) and metabolic endotoxemia. This study investigated the therapeutic effects of inulin, a prebiotic dietary fiber, in attenuating LPS‐induced intestinal epithelial barrier dysfunction. Caco‐2 cells were treated with 100 ng/mL of LPS for 12 h, resulting in increased gene expression of pro‐inflammatory cytokines (IL‐1β, TNF‐α, and IL‐18) and a significant downregulation of tight junction proteins claudin‐1 and claudin‐2, while occludin gene expression remained unaffected. Pretreatment with 2% inulin for 24 h before LPS exposure prevented the downregulation of claudin‐1 and claudin‐2 and significantly upregulated occludin gene expression. These molecular findings were supported by functional assays using transwell systems. LPS treatment increased paracellular permeability of the Caco‐2 monolayer, indicating barrier dysfunction, while inulin pretreatment mitigated this effect. These results demonstrate that inulin can modulate tight junction protein expression and maintain gut barrier integrity under inflammatory conditions.

## Graphical abstract

LPS treatment of Caco‐2 cells significantly increases inflammation in Caco‐2 cells. This increase in inflammation is followed by downregulation of tight junction proteins and increase in paracellular permeability. Inulin pretreatment protects Caco‐2 cells against LPS‐induced damage to the epithelial barrier function.

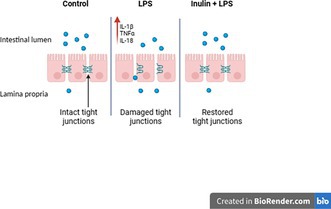

## 1 Introduction

There are over 39 trillion microbes, mostly bacteria, residing in the human gut (Cannon et al. 2016). This diverse microbiome contains both commensal and pathogenic bacteria (Cannon et al. 2016). The relationship between these microorganisms and the host is at times commensal and can prevent or attenuate harmful pathogens from colonizing and infecting the host (Mendler et al. 2020; Nepelska et al. 2017; Kuugbee et al. 2016). Due to the presence of trillions of microbes in the gut, integrity of the gut barrier is of utmost importance (Cannon et al. 2016). The intestinal epithelial barrier serves as a physical and biochemical defense, regulating the transport of nutrients, water, and other substances while preventing the entry of bacteria, toxins, and allergens into the bloodstream. However, when this barrier is compromised, it can result in a leaky gut. The compromised intestinal epithelial barrier is associated with the development of autoimmune diseases, food allergies, and gastrointestinal disorders (Cantorna et al. 2019).

Tight junctions (TJs) are specialized intercellular structures that play a critical role in maintaining the integrity of epithelial and endothelial barriers. Composed of transmembrane proteins such as Claudins, and Occludin, TJs form small, closed ring‐like structures at the apical portion of cell membranes. These structures regulate paracellular permeability, ensuring selective movement of ions and molecules, and maintain cell polarity by segregating apical and basolateral membrane domains (Al‐Sadi et al. 2011; Cording et al. 2013; Horowitz et al. 2023).

Among TJ proteins, Claudins are pivotal due to their diverse roles in forming the backbone of tight junction strands. Claudin‐1 is essential for strengthening the epithelial barrier, while Claudin‐2 exhibits pore‐forming properties, contributing to increased permeability under certain conditions (Cording et al. 2013; Overgaard et al. 2011). Occludin, another integral TJ protein, is primarily involved in stabilizing the tight junction complex and modulating barrier function. Alterations in the expression levels of these proteins are often indicative of compromised epithelial integrity and are linked to various pathological conditions (Al‐Sadi et al. 2011; Horowitz et al. 2023).

In this study, we investigated the expression of Claudin‐1, Claudin‐2, and Occludin as key indicators of tight junction dynamics. These markers were selected because their gene expression reflects changes in epithelial barrier function, providing insights into the molecular mechanisms underlying lipopolysaccharide (LPS)‐induced damage to intestine epithelium. The detection and quantification of these TJ proteins offer valuable information about the impact of LPS and prebiotics on the structural and functional integrity of epithelial barriers.

LPS is found on the cell surface of Gram‐negative bacteria and is a major endotoxin (Reisinger et al. 2020). LPS is a potent inflammation stimulant and is responsible for intestinal and systemic inflammatory reactions that disrupt TJs and induce cytokine secretion (Kordulewska et al. 2020). Pro‐inflammatory cytokines IL‐1β, IL‐6, IL‐8, and TNF‐α are released by leukocytes in response to infection and endotoxemia (Lima et al. 2020). It is proposed that inflammatory signaling molecules and pro‐inflammatory cytokines are responsible for endotoxin‐stimulated gut damage (Wang, Xiao, et al. 2021).

The effects of microbiome dysbiosis are critical and are linked to the reduction of gut microbial diversity (Cantorna et al. 2019). Systemic and intestinal presence of LPS due to increased intestinal permeability has been proposed as the cause of many other diseases outside of IBD and IBS such as: autoimmune diseases, neuroinflammatory disorders, diabetes, coeliac disease, obesity, colorectal cancer, and non‐alcoholic fatty liver disease (Arrieta et al. 2009; Beisner et al. 2021; Cantorna et al. 2019; Wang, Fukui, et al. 2021). Intestine epithelial cell (IEC) damage and systemic LPS exposure may result in sepsis, and systemic inflammation (Reisinger et al. 2020).

Our previous study suggested that anti‐inflammatory agents, such as mesalamine, has the potential to be used as a treatment to reduce intestinal inflammation and restore normal intestinal function following burn injury (Cannon et al. 2016). Research has shown that a lack of dietary fiber leads to decreased microbial diversity and short chain fatty acid (SCFA) production, and a shift towards less desirable substrates like proteins and host mucins (Makki et al. 2018). A handful of investigations have emphasized preventive measures for boosting epithelial barrier integrity in order to reduce gastrointestinal dysfunction (Batra et al. 2021; Holt et al. 2017; Steinberg et al. 2020). One such preventive approach centers on utilizing dietary supplements like prebiotics, given that diet is considered a key factor in shaping the gut microbiota (Lê et al. 2022). Research has indicated that manipulating the gut microbiota through prebiotics might enhance the structure and function of the gastrointestinal system (Lê et al. 2022).

The overall purpose of our current study was to investigate whether inulin treatment can help attenuate LPS‐induced intestine inflammation and gut barrier damage using a Caco‐2 cell model. By exposing Caco‐2 cells to varying concentrations of LPS (1, 10, and 100 ng/mL) for 0, 2, 4, 8, and 12 h, while also pretreating the cells with 2% inulin for 24 h, we have measured the effects of inulin in mitigating IEC dysfunction. We hypothesize that presence of inulin in our diet may play an important role in maintaining IEC health by reducing inflammation and improve TJ protein expression during LPS‐induced barrier dysfunction.

## 2 Materials and Methods

### 2.1 Caco‐2 Cell Culture

Human intestinal Caco‐2 cells were obtained from American Type Culture Collection (ATCC, Manassas, VA) and cultured in T‐75 flasks in Eagle's minimum essential medium (EMEM, Quality Biological, Gaithersburg, MD) supplemented with L‐Glutamine, penicillin (100 units/mL), streptomycin (100 μg/mL), and fetal bovine serum (10%). Cells were incubated at 37°C with 5% CO_2_. The culture medium was changed every 2–3 days, and cells were passaged when confluency reached about 80%–90%. Cells between passages 3 and 6 were used for the experiments.

### 2.2 Cell Treatments

Cells were serum‐starved overnight before any treatment. Cells were treated with LPS (1, 10, and 100 ng/mL; from *E. coli*, Serotype O55:B5) for 0, 2, 4, 8, and 12 h. In the second set of experiments, cells were pretreated with 2% inulin for 24 h before treatment with LPS. Control cells were not treated with either LPS or inulin. At the end of the experimental period, cells were washed with ice‐cold PBS and harvested for total RNA extraction.

### 2.3 RNA Extraction and cDNA Synthesis

The total RNA from Caco‐2 cells was extracted using SV Total RNA Isolation System (Promega, Madison, WI), according to the manufacturer's instructions. An equal amount of purified RNA from each sample was reverse‐transcribed into cDNA using a High‐Capacity RNA‐to‐cDNA Kit (Applied Biosystems, Waltham, MA). cDNA was stored at −20°C for further analysis.

### 2.4 RT‐PCR and Data Analysis

Gene expressions of TJ proteins and pro‐inflammatory cytokines were examined. qPCR was performed in a Biorad CFX96 Real‐Time System using SYBR green PCR master mix (Applied Biosystems, Waltham, MA). A negative control without cDNA was included in each assay. Gene expression was analyzed by Bio‐Rad CFX Maestro software using ΔΔCq method. GAPDH was used as a reference gene to normalize disproportion in the mRNA amount. The results were scaled to the expression level of control which is determined as one.

### 2.5 Determination of Paracellular Permeability

Caco‐2 cells were seeded (5 × 10^5^ cells/well) and cultured in the upper chamber of 6‐well Transwell (Corning Incorporated, New York, NY) insert filters until a monolayer was formed. The cells were then apically treated with either LPS (1, 10, and 100 ng/mL) for 24 h or treated with inulin for 24 h before LPS treatment. Following treatment, the monolayer was washed twice with Phosphate Buffer Saline (PBS) to remove any residual LPS or inulin. For the measurement of apical‐to‐basal paracellular permeability, 1.5 mL of Hanks' Balanced Salt Solution (HBSS) was added to the basal compartment and 0.2 mL of 4 kDa fluorescein isothiocyanate (FITC)‐conjugated dextran (1 mg/mL in HBSS) was added on the apical side of the inserts. After the wells were incubated for 4 h, samples (100 μL) were collected from the basal compartment. Fluorescent intensity was measured using an ELISA reader (BioTek Cytation 5, Agilent Technologies, Santa Clara, CA). The wavelengths of excitation and emission were 490 and 530 nm, respectively.

### 2.6 Statistical Analysis

The data are presented as means + standard error of the mean (SEM) and were analyzed by one‐way analysis of variance (ANOVA) with Tukey's test (SigmaPlot 15.0). *p* < 0.05 was considered statistically significant.

## 3 Results

### 3.1 LPS Treatment Caused an Increase in IL‐1β Gene Expression in Caco‐2 Cells

**FIGURE 1:**
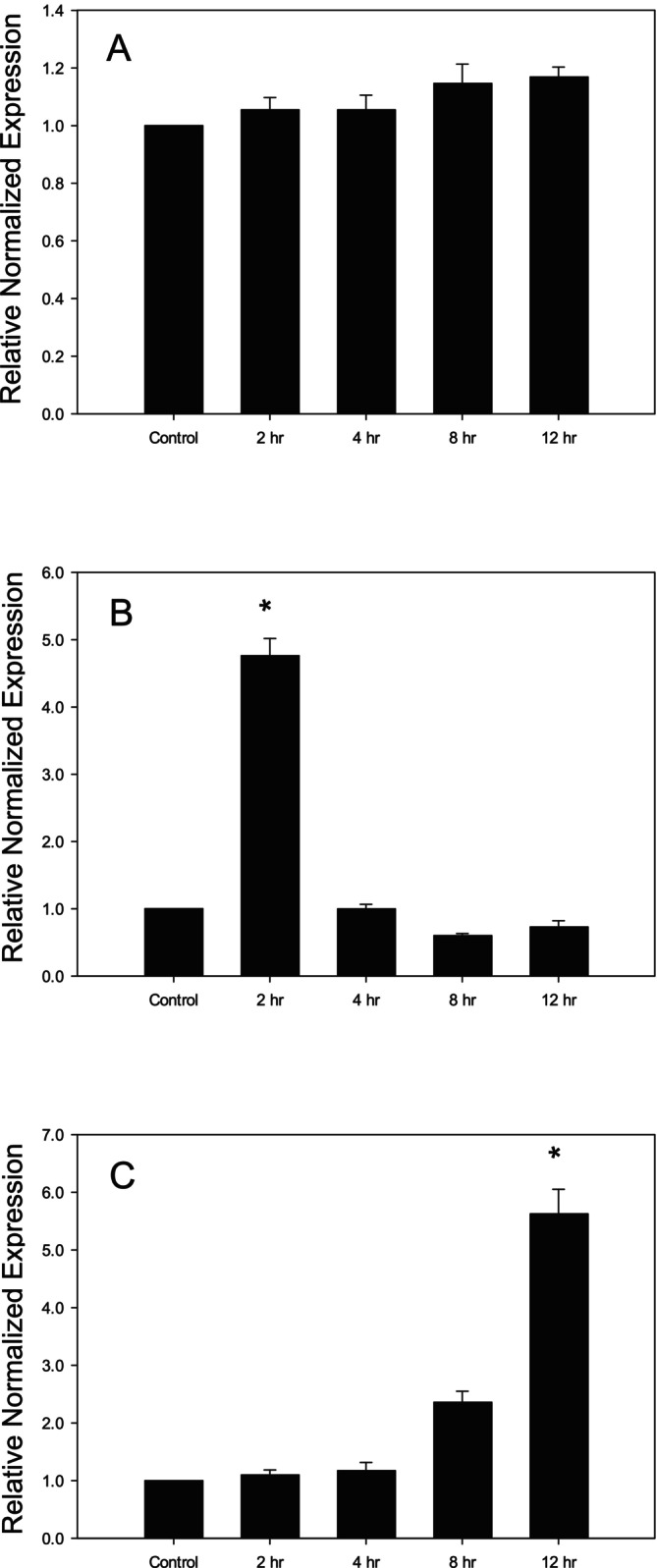
Changes in the gene expression of Il‐1ß in LPS‐treated Caco‐2 cells. (A) IL‐1β gene expression following treatment with LPS (1 ng/mL, 2–12 h), (B) IL‐1β gene expression following treatment with LPS (10 ng/mL, 2–12 h) (C) IL‐1β gene expression following treatment with LPS (100 ng/mL, 2–12 h). *p < 0.05 versus control. Data are reported as mean ± standard error of the mean (SEM) from at least three experiments.

We investigated the effect of different concentrations of LPS (1, 10, and 100 ng/mL) for different time points (0, 2, 4, 8, and 12 h) on IL‐1β gene expression in Caco‐2 cells. As shown in Figure 1A, treatment of Caco‐2 cells with 1 ng/mL (physiologically relevant concentration) of LPS did not cause any change in the gene expression of IL‐1β at any time point. Cells which were treated with 10 ng/mL LPS exhibited a significant increase in IL‐1β gene expression at 2 h. This increase was, however, immediately restored to the control level starting from 4 h (Figure 1B). Treatment of Caco‐2 cells with 100 ng/mL of LPS for 12 h resulted in a significant upregulation of IL‐1β gene expression when compared with the other time points (Figure 1C).

### 3.2 LPS Treatment Caused an Increase in TNF‐α Gene Expression in Caco‐2 Cells

**FIGURE 2:**
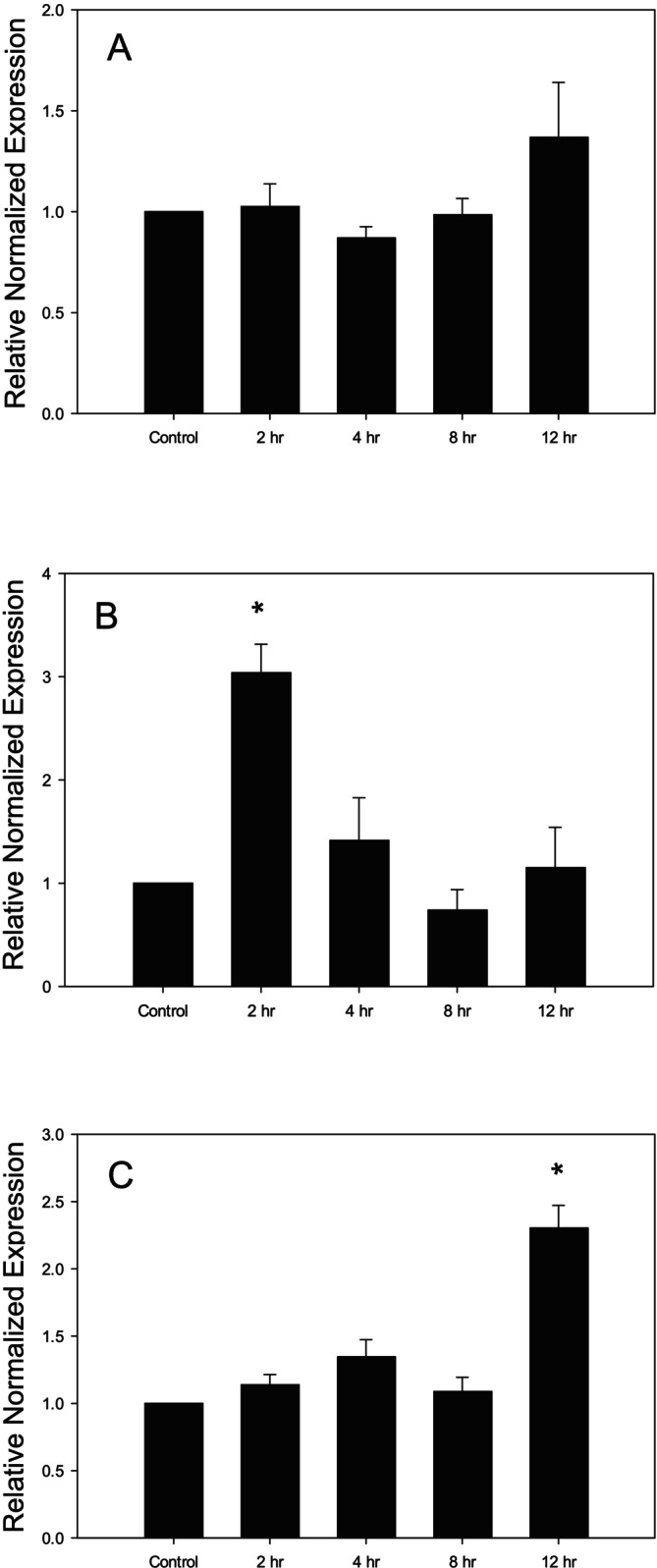
Changes in the gene expression of TNF‐α in LPS‐treated Caco‐2 cells. (A) TNF‐α expression following treatment with LPS (1 ng/mL, 2–12 h), (B) TNF‐α expression following treatment with LPS (10 ng/mL, 2–12 h) (C) TNF‐α expression following treatment with LPS (100 ng/mL, 2–12 h). *p < 0.05 versus control. Data are reported as mean ± standard error of the mean (SEM) from at least three experiments.

When Caco‐2 cells were treated with different concentrations of LPS for different time points, a similar response in TNF‐α gene expression was observed as was seen with IL‐1β gene expression. At any time point, no change in TNF‐α gene expression was observed when Caco‐2 cells were treated with 1 ng/mL of LPS (Figure 2A). There was a significant increase in the gene expression of TNF‐α at 2 h when cells were treated with 10 ng/mL of LPS (Figure 2B). Starting at 4 h, the TNF‐α level was restored to the control level. Treatment of cells with 100 ng/mL of LPS for 12 h resulted in a significant upregulation of TNF‐α when compared with the other time points and the control (Figure 2C).

### 3.3 LPS Treatment Caused an Increase in IL‐18 Gene Expression in Caco‐2 Cells

**FIGURE 3:**
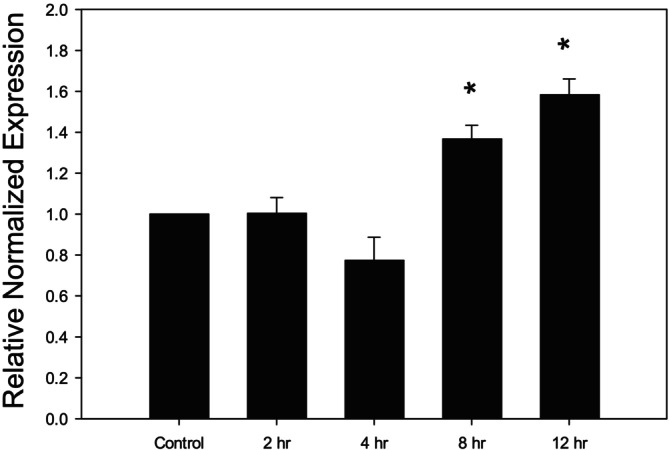
Changes in the gene expression of IL‐18 in LPS‐treated Caco‐2 cells. IL‐18 expression following treatment with LPS (100 ng/mL, 2–12 h). *p < 0.05 versus control. Data are reported as mean ± standard error of the mean (SEM) from at least three experiments.

Since treatment of Caco‐2 cells with 100 ng/mL LPS resulted in a significant upregulation of both IL‐1β and TNF‐α gene expressions, we measured the IL‐18 gene expression after treating the Caco‐2 cells only with 100 ng/mL of LPS for different time points. Compared to control, LPS treatment significantly upregulated the IL‐18 levels both at 8 and 12 h of exposure (Figure 3).

### 3.4 Effect of Inulin Pretreatment on LPS Induced Decrease in TJ Protein Gene Expression

**FIGURE 4:**
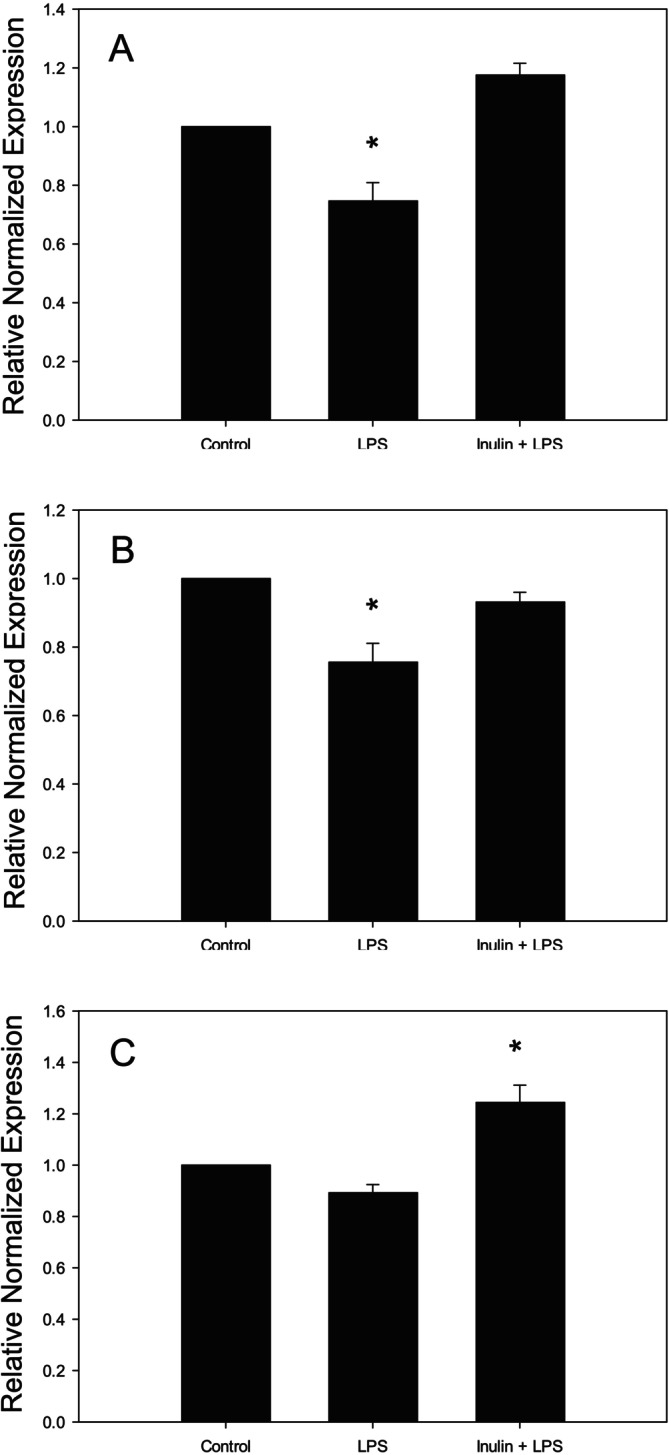
Effect of inulin on tight junction protein gene expression in Caco‐2 cells. (A) Claudin‐1 expression following treatment with LPS (100 ng/mL, 12 h), and with or without inulin pretreatment (2%, 24 h). (B) Claudin‐2 expression following treatment with LPS (100 ng/mL, 12 h), and with or without inulin pretreatment (2%, 24 h). (C) Occludin expression following treatment with LPS (100 ng/mL, 12 h), and with or without inulin pretreatment (2%, 24 h). *p < 0.05 versus other groups. Data are reported as mean ± standard error of the mean (SEM) from at least three experiments.

Next, we pretreated Caco‐2 cells with 2% inulin for 24 h before exposing to LPS to determine whether inulin has any effect on tight junction protein gene expression. As shown in Figure 4A,B, treatment with 100 ng/mL of LPS for 12 h significantly downregulated the gene expression of claudin‐1 and claudin‐2. Pretreatment of Caco‐2 cells with inulin prevented the decrease in the expression of both claudin 1 and 2 almost to the control level. LPS treatment did not change the gene expression of occludin. However, inulin pretreatment significantly increased occludin gene expression beyond the levels of the control (Figure 4C).

### 3.5 Effect of Inulin Pretreatment on LPS Induced Increase in Paracellular Permeability of Caco‐2 Cell Monolayer

**FIGURE 5:**
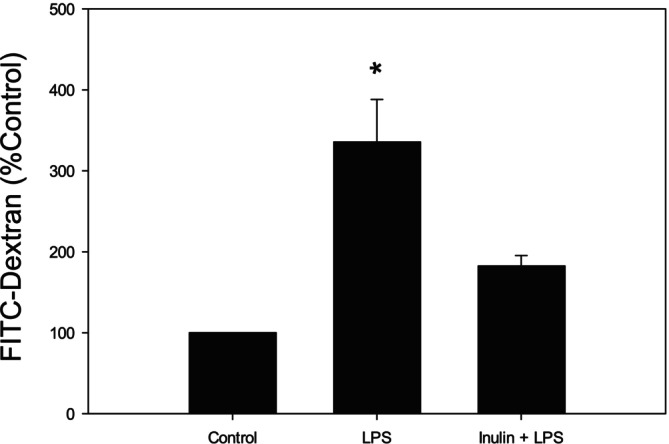
Effect of inulin on the paracellular permeability of Caco‐2 cell monolayer. Paracellular permeability of FITC‐dextran in Caco‐2 cell monolayer treated with LPS (100 ng/mL, 24 h), and with or without inulin pretreatment (2%, 24 h). Four hours after the addition of FITC‐dextran to the apical side of the transwell, samples (100 μL) were collected from the basal compartment and fluorescent intensity was measured. *p < 0.05 versus other groups. Data are reported as mean ± standard error of the mean (SEM) from at least three experiments.

We employed FITC‐dextran diffusion to monitor and quantify paracellular permeability of Caco‐2 cell monolayer. Compared to control, no change in paracellular permeability was observed when Caco‐2 cell monolayer was treated with low doses of LPS (1 and 10 ng/mL). However, when treated with 100 ng/mL of LPS for 24 h, there was a significant increase in solute flux across the Caco‐2 monolayer, indicating a disruption of the epithelial barrier. Pretreatment of Caco‐2 cells with inulin for 24 h mitigated this LPS‐induced increase in paracellular permeability (Figure 5).

## 4 Discussion

Several studies have suggested that fiber‐rich diets have the potential to alleviate intestinal barrier dysfunction (Fritsch et al. 2021; Makki et al. 2018). Inulin, a soluble dietary fiber, possesses prebiotic properties that can modulate the gut microbiota composition and enhance the production of short‐chain fatty acids which promote intestinal barrier function by increasing the expression of tight junction proteins, reducing intestinal permeability, and enhancing mucosal integrity (Bindels et al. 2015; Cani et al. 2007; Everard et al. 2011; Sohail et al. 2017). Moreover, inulin has been found to have anti‐inflammatory effects by suppressing the production of pro‐inflammatory cytokines and modulating immune responses (Bindels et al. 2015; Cani et al. 2007). These combined effects of inulin on the gut microbiota, intestinal barrier integrity, and immune modulation contribute to the potential reduction of intestinal barrier dysfunction (Sohail et al. 2017).

Despite previous studies, further research is needed to fully elucidate the underlying mechanisms and optimal dietary conditions for utilizing inulin as a therapeutic intervention for LPS‐induced inflammation and intestinal barrier dysfunction. In the present study, we were able to demonstrate how exposure to LPS increases Caco‐2 cells inflammation and affects tight junction protein expression and paracellular permeability. Our findings contribute novelty by exploring the potential of inulin dietary fiber in attenuating the disassembly of tight junctions and the increase in paracellular permeability induced by LPS.

Prior studies have demonstrated that exposure to LPS at concentrations up to 80,000 ng/mL does not significantly impact the viability of Caco‐2 cells. However, it does lead to an augmented production of inflammatory cytokines (Fang et al. 2021; Lan et al. 2021; Pistol and Taranu 2021; Serreli et al. 2020). Our study uncovered that low concentrations of LPS (10 ng/mL) minimally induce inflammation, while concentrations at 100 ng/mL exponentially enhance the inflammatory response. Furthermore, we observed that the duration of LPS exposure influences the release of inflammatory cytokines, with longer exposure periods (8–12 h) leading to a significant production of IL‐1β, TNF‐α, and IL‐18.

TNF‐α and IL‐1β are two important inflammatory cytokines involved in LPS‐induced inflammation, but they exhibit some differences in their roles and mechanisms of action. TNF‐α plays a crucial role in the initiation and amplification of the inflammatory response (Aggarwal 2003). It induces the expression of adhesion molecules, activates immune cells, promotes the production of other pro‐inflammatory cytokines, and mediates tissue damage during inflammation (Aggarwal 2003). On the other hand, IL‐1β acts as a key mediator of innate immunity and inflammation (Dinarello 2007).

Interleukins from the IL‐1 family of cytokines, are released into the blood, and surrounding intestinal fluid during inflammation, particularly in individuals diagnosed with active IBD (Al‐Sadi and Ma 2007; Dinarello 2007; Kaneko et al. 2019). In the context of intestinal disorders, IL‐1β acts as a crucial mediator of innate immunity and inflammation, leading to tissue damage (Kaneko et al. 2019). Serving as a master regulator, IL‐1β exerts control over a diverse array of innate immune processes associated with inflammation. Throughout its historical significance, IL‐1β has demonstrated a wide range of biological functions, a mediator of fever and an inducer of several components of the acute‐phase response (Dosh et al. 2019).

The increase in Caco‐2 tight junction permeability caused by IL‐1β was accompanied by rapid activation of NF‐κB (R. M. Al‐Sadi and Ma 2007). NF‐κB inhibitors, pyrrolidine dithiocarbamate and curcumin, were successfully used to prevent the IL‐1β‐induced enhancement of Caco‐2 tight junction permeability. Furthermore, through small interfering RNA transfection, expression of NF‐κB p65 was suppressed, which completely halted the IL‐1β‐induced increase in Caco‐2 tight junction permeability (R. M. Al‐Sadi and Ma 2007). This finding is significant as it demonstrates that the IL‐1β‐induced increase in Caco‐2 tight junction permeability is partly mediated through the activation of NF‐κB pathways rather than apoptosis.

Emerging research suggests that downstream signaling pathways can influence the expression of inflammatory cytokines, which could have a direct impact on the structural composition of Caco‐2 cell monolayers and tight junction proteins. Our investigation on how LPS treatment influences the expression of tight junction proteins in Caco‐2 cells such as claudin‐1, claudin‐2, and occludin expanded the current body of research in this area.

Previous research shows that the structural foundation of tight‐junction strands is formed by claudin‐1 and claudin‐2. These proteins possess four transmembrane domains, but they do not exhibit sequence similarities to occludin (Cording et al. 2013). Differences in molecular sequencing lead to distinct signaling pathways involved in the formation of tight junctions. Protein kinase A (PKA), various forms of protein kinase C (PKC), and monomeric and heterotrimeric G proteins are among the proteins involved in tight junction assembly. The aPKC‐PAR3‐PAR6 complex acts as a promoter for functional tight junctions, while PP2A serves as a negative regulator of tight junctions by dephosphorylating tight junction proteins phosphorylated by a PKC. These opposing signaling pathways, represented by the aPKC‐PAR3‐PAR6 complex and PP2A, play a crucial role in regulating the assembly and disassembly of tight junctions (Weber 2012).

FRAP (Fluorescence Recovery After Photobleaching) experiments have been used to evaluate the assembly of tight junctions by examining the fluorescence recovery over time after selectively photobleaching a fluorescently tagged tight junction protein. The dynamics of this fluorescence displays stability of molecular interactions within the tight junctions (Weber 2012). By comparing the mobility patterns of different tight junction proteins, researchers have been able to infer specific molecular interactions. Under normal conditions, tight junction proteins exhibit constant remodeling and dynamic behavior. A computer model demonstrated distinct behaviors and exchange patterns of these proteins within three cellular compartments: The tight junction, lateral membrane, and cytosol. The model revealed that occludin displayed a rapid recovery rate, wherein regeneration occurred from both the tight junction and lateral membrane compartments (Weber 2012). In contrast, claudin‐1 exhibited a slow recovery, originating from a mobile pool of claudin situated in close proximity to the tight junction (Weber 2012).

Molecular dynamics, alongside other contributing factors, may have influenced the expression of claudin proteins (Figure 4A,B). It is plausible that claudin proteins could exhibit significant expression in Caco‐2 cells that were pretreated with inulin before exposure to LPS conditions, particularly when evaluated over an extended duration (24 h or more). However, further research is necessary to validate this hypothesis.

## Author Contributions

**A. Georgetta Skinner:** data curation (lead). **Abdul Malik:** writing – review and editing (supporting). **M. Rizwan Siddiqui:**review and editing (supporting). **Vineet Singh:** resources (supporting). **Suhail Akhtar:** conceptualization (lead), funding acquisition (lead), investigation (lead), methodology (lead), project administration (lead), supervision (lead), writing – original draft (lead).

## Conflicts of Interest

The authors declare no conflicts of interest.

## Data Availability

The data that support the findings of this study are available from the corresponding author upon reasonable request.
